# Cyclization of the Urokinase Receptor-Derived Ser-Arg-Ser-Arg-Tyr Peptide Generates a Potent Inhibitor of Trans-Endothelial Migration of Monocytes

**DOI:** 10.1371/journal.pone.0126172

**Published:** 2015-05-04

**Authors:** Ali Munaim Yousif, Michele Minopoli, Katia Bifulco, Vincenzo Ingangi, Gioconda Di Carluccio, Francesco Merlino, Maria Letizia Motti, Paolo Grieco, Maria Vincenza Carriero

**Affiliations:** 1 Department of Pharmacy, University Federico II, Naples, Italy; 2 Neoplastic Progression Unit, Department of Experimental Oncology, IRCCS Istituto Nazionale Tumori “Fondazione G. Pascale”, Naples, Italy; 3 SUN Second University of Naples, Italy; 4 University ‘Parthenope’, Naples, Italy; 5 CIRPEB: Centro Interuniversitario di Ricerca sui Peptidi Bioattivi University of Naples “Federico II”, DFM-Scarl, Institute of Biostructures and Bioimaging—CNR, 80134, Naples, Italy; University of Pécs Medical School, HUNGARY

## Abstract

The receptor for the urokinase-type plasminogen activator (uPAR) is a widely recognized master regulator of cell migration and uPAR_88-92_ is the minimal sequence required to induce cell motility. We and others have previously documented that the uPAR_88-92_ sequence, even in the form of synthetic linear peptide (SRSRY), interacts with the formyl peptide receptor type 1 (FPR1), henceforth inducing cell migration of several cell lines, including monocytes. FPR1 is mainly expressed by mammalian phagocytic leukocytes and plays a crucial role in chemotaxis. In this study, we present evidence that the cyclization of the SRSRY sequence generates a new potent and stable inhibitor of monocyte trafficking. In rat basophilic leukaemia RBL-2H3/ETFR cells expressing high levels of constitutively activated FPR1, the cyclic SRSRY peptide ([SRSRY]) blocks FPR1 mediated cell migration by interfering with both internalization and ligand-uptake of FPR1. Similarly to RBL-2H3/ETFR cells, [SRSRY] competes with fMLF for binding to FPR1 and prevents agonist-induced FPR1 internalization in human monocyte THP-1 cells. Unlike scramble [RSSYR], [SRSRY] inhibits fMLF-directed migration of monocytes in a dose-dependent manner, with IC_50_ value of 0.01 nM. PMA-differentiated THP-1 cell exposure to fMLF gradient causes a marked cytoskeletal re-organization with the formation of F-actin rich pseudopodia that are prevented by the addition of [SRSRY]. Furthermore, [SRSRY] prevents migration of human primary monocytes and trans-endothelial migration of monocytes. Our findings indicate that [SRSRY] is a new FPR1 inhibitor which may suggest the development of new drugs for treating pathological conditions sustained by increased motility of monocytes, such as chronic inflammatory diseases.

## Introduction

There is increasing evidence that the urokinase-type plasminogen activator receptor (uPAR) which is expressed by a wide variety of hematologic cells, including monocytes and macrophages, plays an important role in leukocyte recruitment following inflammation [1–3]. Migration of immune cells to tissue lesions is impaired in uPA^−/−^and uPAR^−/−^mice, resulting in impairment of host defenses, bacterial spread, and death [3–7]. Furthermore, plasma levels of soluble forms of uPAR are released in plasma of individuals suffering from viral, bacterial or parasitic infections as well as autoimmune diseases [8–9].

The uPAR is a glycosylated glycosyl-phosphatidyl-inositol (GPI)-anchored protein [10], formed by three domains (DI, DII, and DIII) connected by short linker regions [11]. Enzymatic cleavage with chymotrypsin generates a carboxyl-terminal fragment starting at residue 88 (DIIDIII88–274) that is phenotypically relevant because cleaved forms of uPAR lacking the DI domain are naturally produced and retain the capability to trigger migration and trans-endothelial invasion of cancer cells [12].

uPAR regulates cell migration through the assembly in composite regulatory units with transmembrane receptors including the G-protein coupled formyl peptide receptors (FPR)s and the vitronectin receptor which, in turn, signal across the membranes [13]. Ligand activated uPAR exposes on the cell surface the minimal active ^88^Ser-Arg-Ser-Arg-Tyr^92^ sequence (uPAR_88-92_) which is able to trigger cell migration and angiogenesis *in vitro* and *in vivo*, even in the form of synthetic linear Ser-Arg-Ser-Arg-Tyr peptide (SRSRY) [14–16]. Mechanistically, uPAR_88-92_ sequence interacts with FPRs type 1 and 2, henceforth inducing cell migration of several cell lines including monocytes and macrophages [14–18].

In the last decade, it has been suggested that the multi-domain uPAR may reversibly acquire distinct conformational states that differently impact on its function and that the equilibrium between these conformations may be sensitive to mutations [19–20]. X-ray studies have documented that uPAR_88-92_ sequence, located on the external surface of uPAR, displays some conformational flexibility in both membrane associated and soluble forms of uPAR [21–22]. We have previously shown that residue Ser^90^ is positioned in a critical “hinge”, which possibly influences the conformation of nearest residues. Indeed, the substitution of Ser90 in full length, membrane-associated uPAR with a glutamic acid residue prevents the complex uPAR/FPR1/vitronectin receptor cross-talk, thereby blocking tumour cell migration and invasion *in vitro* and *in vivo* [23]. Furthermore, synthetic linear peptides carrying Ser90 to Glu or Cα-methyl-α-aminoisobutyric acid (Aib) substitutions potently inhibit *in vitro* and *in vivo* cell migration, invasion and/or angiogenesis [24–27].

Based on this information, we reasoned that cyclization of the Ser-Arg-Ser-Arg-Tyr peptide could reduce conformational flexibility of its linear form, thus generating a new, more stable peptide that could regulate uPAR_88-92_-dependent functions. This study was aimed to investigate the effects of cyclic Ser-Arg-Ser-Arg-Tyr peptide ([SRSRY]) on the motility of monocytes. We document that both linear SRSRY and [SRSRY] peptides compete with fMLF for binding to FPR1, thus preventing agonist-induced FPR1 internalization in THP-1 cells. However, these peptides exert opposite effect on monocyte motility, the linear SRSRY promotes cell migration, while the peptide [SRSRY] inhibits cell migration in a dose-dependent manner, with IC_50_ value of 0.01 nM. Finally, we show that [SRSRY] prevents trans-endothelial migration of monocytes and causes a marked inhibition of cytoskeletal re-organization occurring during locomotion.

## Results

### Synthesis, time-dependent stability and HPLC profile of [SRSRY] peptide in human serum

**Fig 1:**
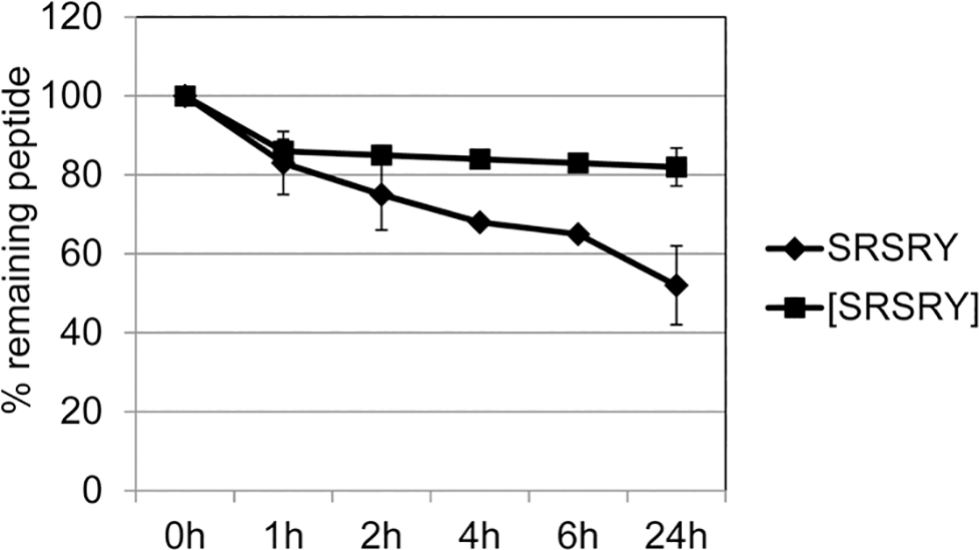
Stability of SRSRY and [SRSRY] peptides in human serum. The stability of [SRSRY] and SRSRY was performed by the injection of the peptide at a 10^–2^ mol/L in human serum at 37°C. Intervals of analysis by RP-HPLC on the compound incubated in serum were carried out growing with time from 1 to 24 hours. Values are expressed as a percentage of peptide concentration assessed at time 0. [SRSRY] (■) make evident its human serum stability respect SRSRY(♦) by RP-HPLC analysis.

Cyclization of peptides listed in **Table 1**was obtained as described in the Material and Methods, using the strategy depicted in the **S1 Fig.** The stability of the linear peptide Ser-Arg-Ser-Arg-Tyr (SRSRY) and cyclized peptide Ser-Arg-Ser-Arg-Tyr [SRSRY] in human serum was investigated through incubation of the peptide at a 10^–2^ mol/L in human serum and samples were taken at different time points and analysed by Reversed-phase chromatography (RP-HPLC). Serum-dependent degradation of peptides was monitored for up to 24 hours. The experiments revealed a gradual degradation of SRSRY through serum protease over time, whereas [SRSRY] retained an evident higher stability. As shown in **Fig 1**, after one hour of exposure to serum, SRSRY and [SRSRY] peptides had a residual concentration lower than 75% and 85%, respectively. After 24 hours, [SRSRY] showed a residual concentration higher than 80% while for compound SRSRY the concentration was lower 50% (**S2 Fig**).

**Table 1:** Synthetized peptides.

Peptide	Sequence	Molecular Weight
SRSRY	H-Ser-Arg-Ser-Arg-Tyr-NH_2_	667.36
[SRSRY]	[Ser-Arg-Ser-Arg-Tyr]^§^	650.20
[RSSYR]	[Arg-Ser-Ser-Tyr-Arg] ^§^	650.20

^§^head-to tail cyclization

### Binding of SRSRY and [SRSRY] peptides to FPR1

**Fig 2:**
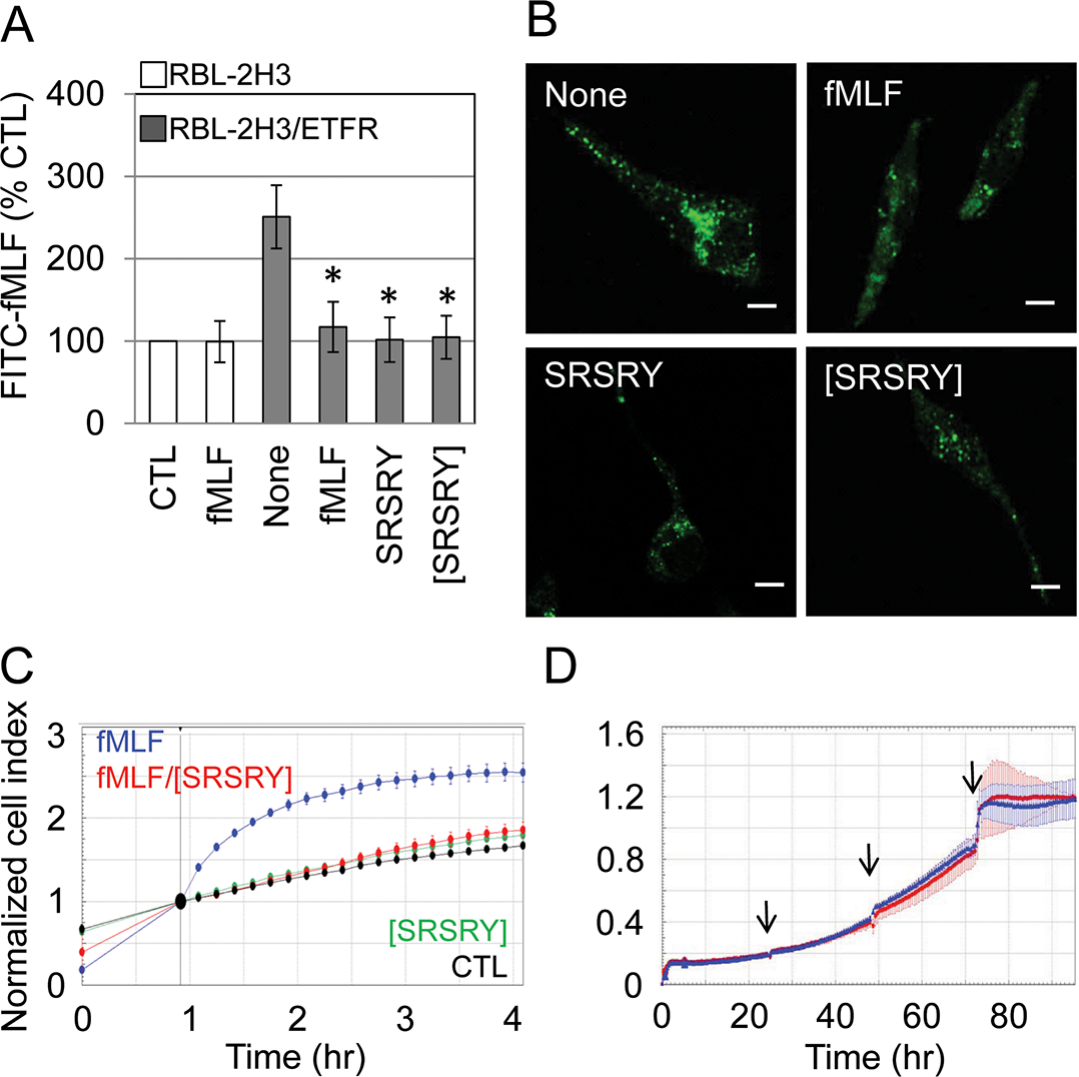
Both linear and cyclic SRSRY peptides bind to FPR1. A. RBL-2H3 or RBL-2H3/ETFR cells (1.5 x10^6^ cells/sample) pre-incubated with diluents (None), 100 nM fMLF, 100 nM SRSRY or 100 nM [SRSRY] for 60 minutes at 4°C and then exposed to diluents or 10 nM N-formyl-Nle-Leu-Phe-Nle-Tyr-Lys-fluorescein (FITC-fMLF) for 60 minutes at 4°C. Fluorimetric measurement of cell-associated fluorescence was assed using 485 nm excitation and 535 nm emission filters. Data are expressed as a percentage of the fluorescence associated to RBL-2H3 cells exposed to FITC-fMLF alone (CTL = 100%) and represent a mean ± SD from triplicates. *Statistical significance (t-test) against None with p<0.001. B. Representative images of RBL-2H3/ETFR cells incubated with diluents (None), 100 nM fMLF, 100 nM SRSRY or 100 nM [SRSRY] for 30 minutes at 37°C and exposed to 10 nM FITC-fMLF for additional 30 minutes at 37°C. Scale bar: 5 μm. Original magnifications: 630x. C. Cell migration of RBL-2H3/ETFR cells monitored by the xCELLigence system. Cells were seeded on CIM-16-well plates and allowed to migrate toward DMEM (CTL), 10 nM [SRSRY], 10 nM fMLF or 10 nM fMLF mixed with 10 nM [SRSRY]. Cell migration was monitored in real-time for 4 hours as changes in Cell Index. Values were normalized immediately after cell addition. Data represent mean ± SD from a quadruplicate experiment. D. Cell proliferation of RBL-2H3/ETFR cells assessed by monitoring impedance by RTCA xCELLigence system. Growth medium with (red) or without 10 μM [SRSRY] (blue) was replaced every 24 hours (arrows). Data represent mean ± SD from a quadruplicate experiment.

We have previously documented that SRSRY competes with the bacterial-derived N-formyl-methionyl-leucyl-phenylalanine peptide (fMLF) for binding to FPR type 1 (FPR1) and that both SRSRY and fMLF promote cell migration by triggering FPR1 internalization [15, 28]. To test whether [SRSRY] binds to FPR1, RBL-2H3 cells which do not express FPR1 and RBL-2H3/ETFR cells stably expressing FPR1 [24–25], were pre-incubated with diluents, an excess (100 nM) of peptides fMLF, SRSRY, or [SRSRY] for 60 minutes at 4°C (to avoid internalization), and then exposed to 10 nM N-formyl-Nle-Leu-Phe-Nle-Tyr-Lys-fluorescein (FITC-fMLF) for additional 60 minutes at 4°C. Quantification of cell-associated fluorescence was assessed by reading cell lysates with a fluorescence plate reader. RBL-2H3 cells lack FPR1, thus they display very low uptake that was not saturable with unlabeled fMLF (**Fig 2A**). In contrast, FPR1 expressing RBL-2H3/ETFR cells exhibit a specific binding that was reduced to the basal level by unlabelled fMLF as well as SRSRY, as expected. We found that the peptide [SRSRY] inhibits binding of fluorescent agonist to RBL-2H3/ETFR cells to a similar extent as compared to fMLF or SRSRY (**Fig 2A**). To evaluate the effect of [SRSRY] on agonist-dependent FPR1 internalization, binding experiments were performed at 37°C. Upon exposure of RBL-2H3/ETFR cells to FITC-fMLF, FPR1 appeared mainly internalized as indicated by punctuate green fluorescent intra-cytoplasmic spots which were prevented by cell pre-incubation with 100 nM fMLF or 100 nM SRSRY, as expected. An excess of [SRSRY] prevented agonist-dependent FPR1 internalization (**Fig 2B**). Thus, we assessed whether [SRSRY] elicits effects on fMLF-directed cell migration. RBL-2H3/ETFR cell migration toward 10 nM fMLF was monitored in real-time for 4 hours as Cell Index changes due to the adhesion of migrating cells to microelectrodes using the xCELLigence RTCA technology as previously described [12]. In the presence of 10 nM [SRSRY] alone, cell migration did not change significantly. In contrast, 10 nM fMLF elicited a considerable RBL-2H3/ETFR cell migration that was reduced to the basal level by the addition of 10 nM [SRSRY] (**Fig 2C** and **S3 Fig**). The inhibitory effect on migration is not due to a reduced proliferation rate, because 10 μM [SRSRY] did not modify cell growth up to 90 hours, as shown by proliferation curves automatically monitored with the xCELLigence system (**Fig 2D** and **S4 Fig**). These findings indicate that SRSRY is a ligand of FPR1, also when it is in a cyclized form though exerts an opposite effect on cell migration.

### Effect of the peptide [SRSRY] on the motility of monocytes and macrophages

**Fig 3:**
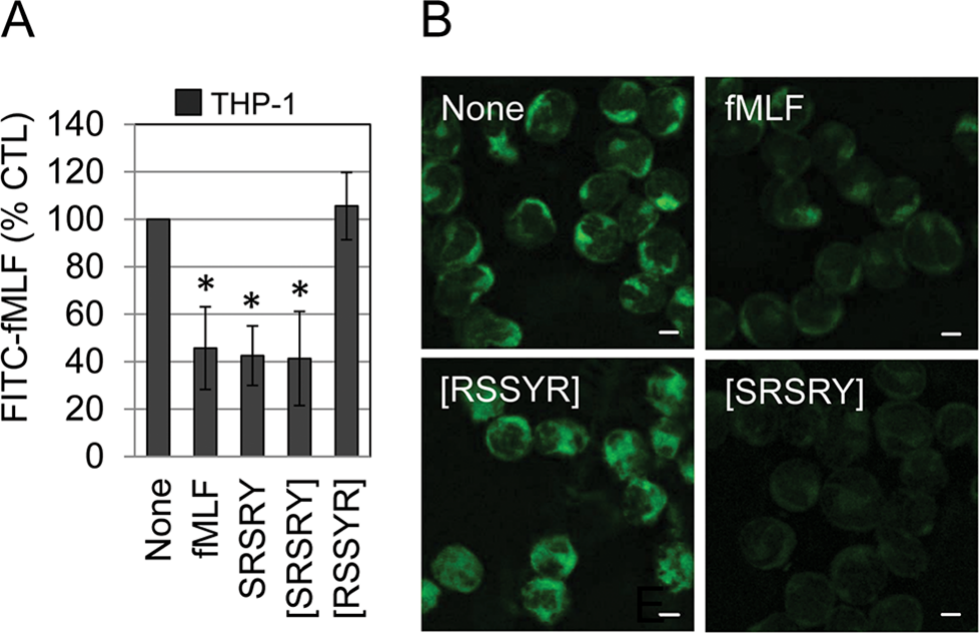
The peptide [SRSRY] binds to FPR1 on THP-1 cell surface. A. THP-1 cells (1.5 x10^6^ cells/sample) were pre-incubated with diluents (None), 100 nM fMLF, 100 nM SRSRY or 100 nM [SRSRY] peptides for 60 minutes at 4°C, then exposed to 10 nM N-formyl-Nle-Leu-Phe-Nle-Tyr-Lys-fluorescein (FITC-fMLF) for additional 60 minutes at 4°C. Fluorimetric measurement of cell-associated fluorescence was assed using 485 nm excitation and 535 nm emission filters. Data are expressed as a percentage of the fluorescence associated to THP-1 cells exposed to FITC-fMLF alone (None = 100%) and represent a mean ± SD from triplicates. *Statistical significance (t-test) against None with p<0.001. B. THP-1 cells incubated with diluents (None), 100 nM fMLF, 100 nM SRSRY or 100 nM [SRSRY] peptides for 30 minutes at 37°C and exposed to 10 nM FITC-fMLF for additional 30 minutes at 37°C. Scale bar: 5 μm. Original magnifications: 630x.

Once documented that both SRSRY and [SRSRY] peptides compete with fMLF for binding to FPR1, we investigated the effect of [SRSRY] on the motility of monocytes. Preliminarily, we assessed whether [SRSRY] is a ligand of FPR1 on monocyte cell surface. Similarly to RBL-2H3/ETFR cells, THP-1 cells exposed to 10 nM FITC-fMLF at 4°C, revealed an appreciable binding which was dramatically reduced by an excess of unlabeled fMLF, SRSRY or [SRSRY], but not by the control peptide [RSSYR] (**Fig 3A**). When binding experiments were performed at 37°C, FPR1 appeared mainly localized within the cytoplasm, adjacent but outside the nucleus of THP-1 cells. Internalization of fluorescent agonist was dramatically reduced in all cell population when THP-1 cells were pre-incubated with an excess of unlabelled fMLF, or [SRSRY] while the control peptide [RSSYR] did not exert such effect (**Fig 3B**). Although we did not determine the binding affinity of [SRSRY] for FPR1, our findings indicate that the peptide [SRSRY] prevents fMLF/FPR1 interaction and inhibits agonist-induced FPR1 internalization in THP-1 cells.

**Fig 4:**
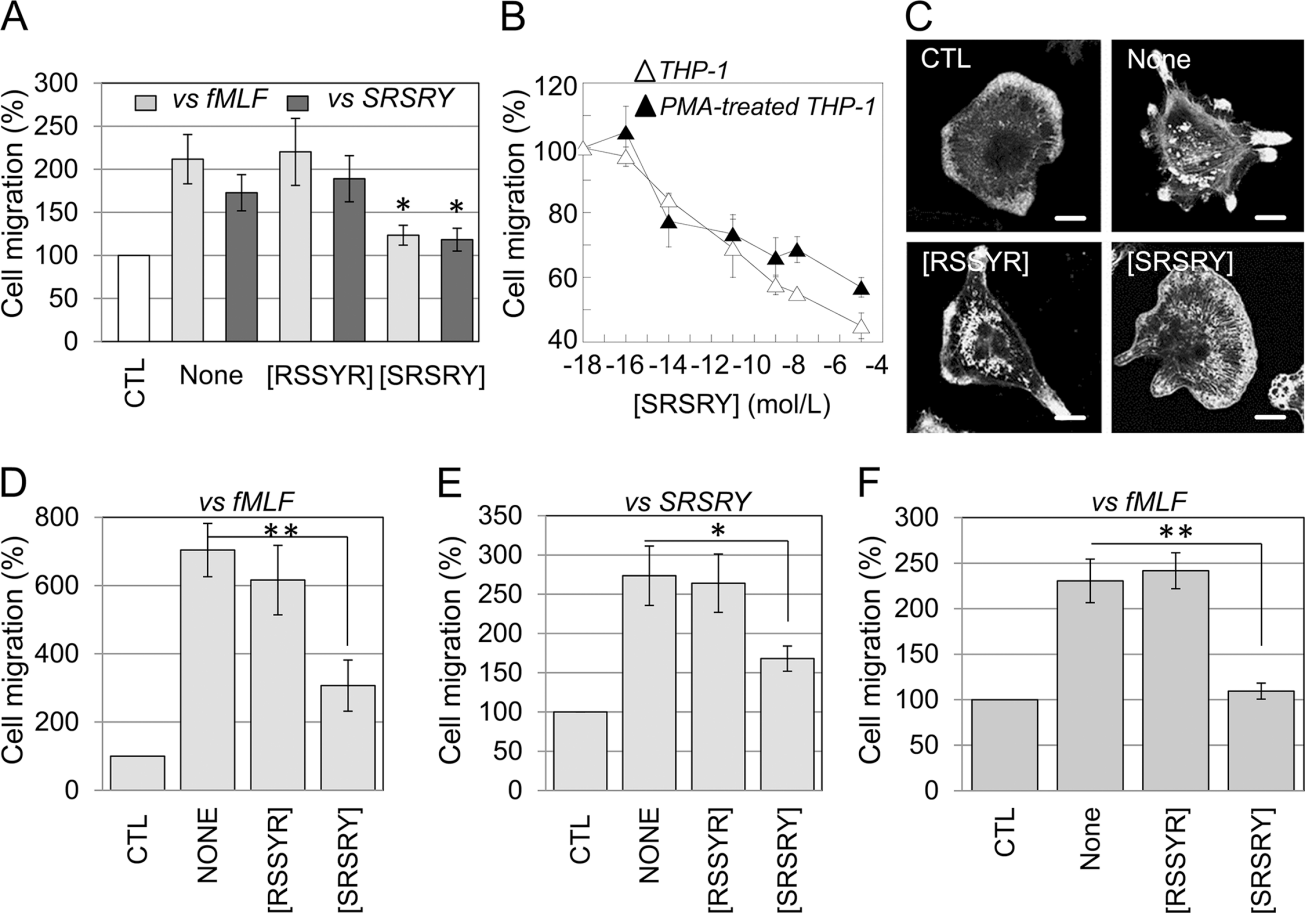
[SRSRY] inhibits migration of monocytes and macrophages in a dose-dependent manner causing a marked inhibition of fMLF-dependent cytoskeletal re-organization. A. THP-1 cells (1x10^5^ cells/well) were allowed to migrate for 90 minutes at 37°C, 5% CO_2_ in Boyden chambers toward 10 nM fMLF, or 10 nM SRSRY in the presence of diluents (None), 10 nM [RSSYR] or 10 nM [SRSRY]. The arbitrary value of 100% was given to the basal cell migration, assessed in the absence of chemoattractant (CTL) and the results were expressed as percentage of the basal cell migration. Data are expressed as the means ± SD of three independent experiments, performed in duplicate. *Statistical significance against None with p<0.001. B. THP-1 and PMA-stimulated THP-1 cell migration toward 10 nM fMLF plus increasing concentrations of [SRSRY] in Boyden chambers. The extent of cell migration was expressed as a percentage of the net fMLF-dependent cell migration, considered as 100%. Data are expressed as the means ± SD of three independent experiments, performed in duplicate. C. Representative images of PMA-stimulated THP-1 cells allowed to migrate in a DUNN chamber toward diluents (CTL), or a fMLF chemotactic gradient, in the absence (None) or presence of 10 nM [SRSRY] or [RSSYR] peptides and stained with rhodamine-phalloidin. Scale bar: 10 μm. Original magnifications: 630x. D-E. Primary monocytes (1x10^5^ cells/well) were allowed to migrate for 90 minutes at 37°C, 5% CO_2_ in Boyden chambers toward 10 nM fMLF (D), or 10 nM SRSRY (E) in the presence of diluents (None), 10 nM [RSSYR] or 10 nM [SRSRY]. The arbitrary value of 100% was given to the basal cell migration, assessed in the absence of chemoattractant (CTL) and the results were expressed as percentage of the basal cell migration. Data are expressed as the means ± SD of three independent experiments, performed in duplicate. *Statistical significance against None with p<0.001; **Statistical significance against None with p<0.0001. F. Primary macrophages allowed to migrate in a DUNN chambers toward diluents (CTL), or a 10 nM fMLF gradient, in the absence (None) or presence of 10 nM [SRSRY] or 10 nM [RSSYR] peptides. For quantitative analysis, a total of 100 macrophages/sample that translocated to the area corresponding to the outer well were counted with an inverted microscope. The arbitrary value of 100% was given to the basal cell migration, assessed in the absence of chemoattractant (CTL) and the results were expressed as percentage of the basal cell migration. *Statistical significance with p<0.001. **Statistical significance with p<0.0001.

The ability of [SRSRY] peptide to affect THP-1 cell migration was analyzed in Boyden chambers using 10 nM fMLF or 10 nM SRSRY as chemoattractants. Not surprisingly, 10 nM fMLF and SRSRY elicited a considerable cell migration, reaching 211% and 172% of the basal cell migration (CTL), respectively. [SRSRY] at 10 nM concentration, reduced monocyte migration toward 10 nM fMLF or 10 nM SRSRY by 58% and 68%, respectively, whereas [RSSYR] did not exert any effect (**Fig 4A**). Inhibitory effect of [SRSRY] is dose-dependent, it starts in the high fM range, it seems to level off in the μM range and reaches an overall 50% reduction at 10 pM (**Fig 4B**). Interestingly, a dose-dependent inhibition is exerted by [SRSRY] also on migration of PMA-differentiated THP-1 cells with IC_50_ value 10 pM (**Fig 4B**). The mechanisms by which monocytes move when subjected to a chemoattractant gradient, involve changes in cytoskeletal organization, which provide both protrusive and contractile forces necessary for cell migration. To gain some insights into the cellular effects exerted by peptide [SRSRY], PMA-differentiated THP-1 cells were grown adherent onto a glass slide, exposed to diluents (CTL), 10 nM fMLF gradient plus/minus 10 nM [SRSRY] or 10 nM [RSSYR] in a DUNN chamber for 6 hours as described [25], and then stained with rhodamine-phalloidin. A total of 100 cells/sample that translocated to the area corresponding to the ring separating the inner and outer chambers were counted and their cytoskeletal organization examined with a fluorescence-inverted microscope. Cells subjected to the fMLF gradient alone (None) or mixed with [RSSYR] control peptide exhibited an elongated morphology and recognizable aligned protrusions associated to locomotion in the 70% cell population. Conversely, the addition of peptide [SRSRY] to the fMLF gradient reduced cell elongation and alignment with the appearance of F-actin linear distribution along the plasma membranes in the 60% of cell population (**Fig 4C**). In order to verify whether the inhibitory effect of [SRSRY] also occurs on primary monocytes and/or macrophages, human monocytes and macrophages were subjected to cell migration assays in Boyden and DUNN chambers, respectively. As shown in **Fig 4**, 10 nM fMLF (**panel D**) or 10 nM SRSRY (**panel E**) elicited a considerable monocyte migration, reaching 644% and 273% of the basal cell migration (CTL), respectively. Unlike [RSSYR], 10 nM [SRSRY] reduced both fMLF- and SRSRY- directed monocyte migration by 50% and 49%, respectively. Next, primary macrophages were subjected to cell migration assay in DUNN chamber as above described. After 6 hours incubation, a total of 100 cells/sample that translocated to the area corresponding to the ring separating the inner and outer chambers were counted. Macrophage exposure to 10 nM fMLF elicited a considerable cell migration, reaching 230% of the basal cell migration (CTL), that was unchanged by 10 nM [RSSYR] (**Fig 4F**). On the contrary, the addition of 10 nM [SRSRY] to the fMLF gradient, reduced migration of primary macrophages to the basal level (**Fig 4F**). These findings indicate that unlike linear SRSRY, the cyclic form of the chemotactic sequence of uPAR inhibits monocyte motility and prevents cytoskeletal re-organization occurring during migration of macrophages.

### Effect of the peptide [SRSRY] on trans-endothelial migration of THP-1 cells

**Fig 5:**
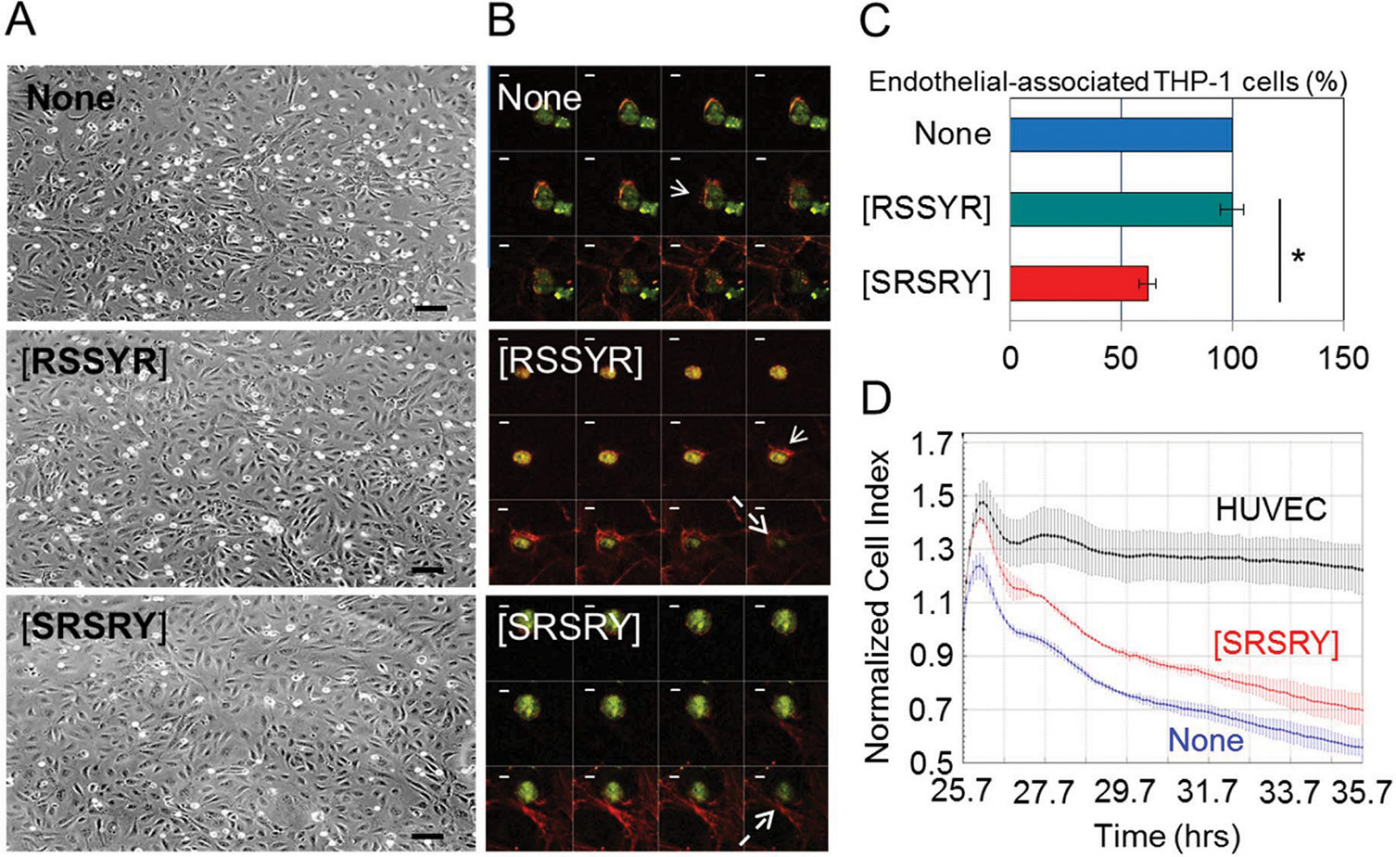
[SRSRY] prevents trans-endothelial migration of THP-1 cells. A-C. Effects of [SRSRY] on the morphology and number of THP-1 adhered to endothelial monolayer. HUVECs (5x10^4^/well) were seeded on matrigel coated coverslips and allowed to adhere for 3 hours, prior to adding GFP-tagged THP-1 cells (1x10^4^ cells/well) exposed to diluents, or the indicated peptides at 10 nM concentration in complete endothelial medium for 30 minutes at 37°C. Representative images of co-cultures analyzed by phase contrast microscopy at 100x with Scale bar: 50 μm (A) or stained with rhodamine- phalloidin and recorded by a confocal microscope (z-series with 0.25 μm intervals) at 630x with Scale bar: 10 μm (B). Arrows: F-actin rich lamellipodia; dotted arrows: THP-1 cells underneath the endothelium. C. Fluorescent THP-1 cells which were adherent on the endothelial monolayer were counted on 10 fields/slide at 200x magnification. Data are expressed as the percentage of the endothelial-associated GFP-THP-1 cells in the absence of peptides (None = 100%).*Statistical significance with p<0.001. D. HUVECs (1x10^4^ cells/well) were seeded in E-plates and allowed to o adhere for ~24 hours until they form a confluent monolayer, prior to seeding THP-1 cells (1x10^4^ cells/well) in the presence of 10% FBS plus/minus 10 nM [SRSRY]. Endothelial invasion by THP-1 cells was monitored in real-time for 10 hours as changes in Cell Index. Values were normalized immediately after THP-1 cell addition. Data represent mean ± SD from a quadruplicate experiment.

Diapedesis of leukocytes plays a key role in the pathogenesis of inflammatory diseases and migration of monocytes from the blood into the sub-endothelial space is one of the earliest events [29–30]. We investigated the effects of [SRSRY] on the number and morphology of THP-1 seeded to an endothelial monolayer. Phase-contrast images revealed numerous THP-1 cells interacting with HUVECs, that decreased upon addition of 10 nM [SRSRY] (**Fig 5A**). To examine the changes in morphology in more detail and quantify monocytes interacting with endothelial monolayer, we performed a subset of experiments using GFP-tagged THP-1, labeling co-cultures for F-actin and recording images by a confocal microscope. In the presence of diluents, or [RSSYR], GFP-tagged THP-1 cells formed F-actin rich lamellipodia and pseudopodia (arrows) which disappeared when GFP-tagged THP-1 were exposed to 10 nM [SRSRY] (**Fig 5B**). Z-stack analysis of confocal images revealed transmigration of THP-1 cells underneath the endothelium (dotted arrows) (**Fig 5B**). In contrast, when the peptide [SRSRY] was added to the co-culture, the majority of THP-1 were seen to rest on top of endothelial cells (**Fig 5B**). Also, a 40% reduction of GFP-THP-1 cell number interacting with endothelial monolayer was achieved by the addition of 10 nM [SRSRY] **(Fig 5C)**. These findings indicate that [SRSRY] not only prevents monocyte interaction with endothelium but also reduces their trans-endothelial migration. To further ascertain if [SRSRY] reduces trans-endothelial migration of monocytes, the ability of THP-1 cells to cross an endothelial monolayer was analyzed using the xCELLigence RTCA technology as previously described [12]. HUVECs were allowed to grow until they formed a monolayer for 24 hours prior to seeding THP-1 cells in the presence of 10% FBS plus/minus [SRSRY]. At this time, reduction of impedance values, due to invading cells that interrupt monolayers was monitored in real-time for 10 hours. As shown in **Fig 5D** and **S5 Fig**, THP-1 cells were able to cross endothelial monolayers and a 35% reduction of endothelial monolayer integrity was achieved by the addition of 10 nM [SRSRY].

Taken together, these data indicate that FPR1-mediated [SRSRY] inhibitory effect involves a marked inhibition of cytoskeletal re-organization occurring during locomotion and trans-endothelial migration of monocytes.

## Discussion

Migration of leukocytes across the wall of blood vessels into tissues is one of the key events during inflammation, immune response against infection and tissue remodeling, representing a key process in the pathogenesis of chronic inflammatory diseases. To date, several new targets and inhibitors that mediate leukocyte motility are being developed [31–32]. However, currently available therapies are often not efficient enough or may even induce undesired effects [33].

uPAR plays an important role in the regulation of leukocyte trafficking [34]. We and others have shown that the capability of uPAR to trigger cell migration depends on its ^88^Ser-Arg-Ser-Arg-Tyr^92^ chemotactic sequence even in the form of a synthetic, linear peptide (SRSRY). Herein we provide evidence that the cyclization of the ^88^Ser-Arg-Ser-Arg-Tyr^92^ chemotactic sequence of uPAR generates a new peptide [SRSRY] exerting an opposite effect on cell migration, as compared to its linear form. [SRSRY] is a new potent inhibitor of monocyte locomotion with IC_50_ value of 0.01 nM. Moreover, [SRSRY] displays higher resistance to enzymatic digestion as compared to other uPAR-derived peptide inhibitors [24].

We have previously reported that, similarly to fMLF, SRSRY promotes cell migration upon interaction with the G protein-coupled FPR1 [14–15, 18]. SRSRY triggers FPR1 activation by inducing its internalization. [16]. Now, by competition experiments with a fluorescent fMLF structures analogue we show that an excess of [SRSRY] competes with fMLF and SRSRY for binding to FPR1, and prevents agonist-induced FPR1 internalization. Our findings suggest that the linear and cyclic structures of the uPAR chemotactic sequence could share the same binding site on FPR1, although they exert opposite effects on cell motility. Alternatively, as the ligand binding pocket of FPR1 consists of several key residues located in different trans-membrane helices [29, 35–36], it is possible that [SRSRY]/FPR1 association may affect SRSRY and fMLF adjacent binding sites. Regarding the specific affinity of [SRSRY] for its target, labeled [SRSRY] is unavailable to us, and therefore we could not determine the affinity of [SRSRY] for FPR1. How [SRSRY] interacts with FPR1 remains to be investigated. In this respect, [SRSRY] could act as an inverse agonist by shifting, upon binding to FPR1, the active state of the receptor toward the inactive one. This issue is relevant *in vivo* for potential therapeutic applications, since inverse agonist effects are associated with receptor activation and inactivation, whereas neutral antagonists produce no effect when administered alone, but blocks the effects of agonists and inverse agonists [37]. Previously, by analysing conformational preference of the sequence Arg-X_1_-Arg-Tyr in the Protein Data Bank, we found that the Arg-Ser-Arg-Tyr sequence shows an equal distribution among the α-turned, β-extended, or random conformation, whereas the Arg-Glu-Arg-X_2_ is mainly observed in α-turn conformation; we also found that the synthetic peptide Arg-Glu-Arg-Phe (RERF) adopt an α-turn conformation and revealed to be a strong inhibitor of cell migration [24]. Since RERF is expected to be susceptible to proteolytic degradation, a series of N-acetylated and C-amidated peptide analogues were designed and characterized for their biochemical and biological properties. Among these, UPARANT which inhibits VEGF-driven angiogenesis in vitro and in vivo more efficiently than RERF, revealed to be stable in blood having a long-time resistance to enzymatic digestion [27]. However, although both RERF and UPARANT adopt in solution a helicogenic turned structure, UPARANT adopts a more compact turned structure with respect to RERF [24, 27]. This may explain some functional differences between RERF and UPARANT as UPARANT seems to have an additional, unidentified binding site on cell surfaces (unpublished). The cyclic peptide [SRSRY] was designed with the aim to develop a stable and more specific inhibitor of the uPAR/FPR1 interaction. Next step will be to analyze the conformational preferences of the peptide [SRSRY]. It could be envisaged that cyclization confers a structural constraint, forcing the molecule to assume a conformation similar to that described for RERF. This issue is relevant *in vivo* for potential therapeutic applications, since a reduced conformational flexibility may enhance potency, selectivity, stability and bioavailability as well as membrane barrier permeability.

Since monocytes express FPR2 and the linear peptide SRSRY is able to bind also FPR2 [14, 38], it will be interesting to ascertain whether a direct [SRSRY]/FPR2 interaction does occur. If this is the case, it could be envisaged the employment of [SRSRY] to counteract chronic inflammatory diseases sustained by continued oxidative stress, including cancer, diabetes, cardiovascular, neurological and pulmonary diseases [38–39].

Activation of FPRs results in increased cell migration, phagocytosis, release of pro-inflammatory mediators, and the signaling cascade culminates in heterologous desensitization of other receptors including chemokine receptors CCR5 and CXCR4, [39]. uPAR expression regulates the adhesive and migratory ability of CXCR4-expressing cells through a mechanism involving FPR1 [40]. Thus, by interacting with a variety of exogenous and host-derived agonists, FPRs constitute a novel group of pharmacological targets.

### Conclusions

Given the finding that peptide [SRSRY] inhibits cell migration by antagonizing FPR1 biologic activity, [SRSRY] may be considered as a potent and stable FPR1 inhibitor which may suggest the generation of new pharmacological treatments for diseases sustained by an excess of monocyte trafficking, such as chronic inflammatory diseases and cancer.

## Materials and Methods

### Peptide synthesis and purification

Linear peptide SRSRY was synthesized by Fmoc chemistry solid-phase approach, purified by RP-HPLC to 99% purity and sequence was verified correct by mass spectrometry as previously described [24]. The synthesis of cyclic peptides [SRSRY] and [RSSYR] listed in Table 1, was carried out using a 2-chlorotritylchloride resin (0.312 g, 0.8 mmol/g), prior swelled for 40 min in DCM. The first coupling was carried out by adding *N* ^α^-Fmoc-Tyr(*t*Bu)-OH (1.0 equiv) dissolved in 5 mL of DCM to the resin. DIPEA (1.0 equiv) was added to this mixture that was stirred in an automated shaker for 10 min. Then, DIPEA (1.5 equiv) was added again. The mixture was agitated vigorously for 1 h at rt. To endcap any remaining unreacted 2-chlorotritylchloride groups, a mixture of DCM/MeOH/DIPEA (80:15:5) was added and mixed for 1 h. To remove Fmoc from the first amino acid, the resin was suspended in 25% piperidine solution in DMF (1 x 5 min and 1 x 25 min). The following protected amino acids were then added stepwise, *N* ^α^-Fmoc-Ser(*t*Bu)-OH and *N* ^α^-Fmoc-Arg(Pbf)-OH. Each coupling reaction was accomplished using a 3-fold excess of amino acid with HBTU (3 equiv) and HOBt (3 equiv) in the presence of DIPEA (6 equiv). The N-terminal Fmoc protecting group was removed as described above. The resin was washed with DMF (3x) and DCM (3x) and dried *in vacuo*. The peptide was released from the resin by a mixture of DCM/AcOH/TFE (80:10:10) for 1 h. The resin was removed by filtration and linear crude peptides were recovered by precipitation in chilled diethyl ether to give pale yellow powders. Head-to-tail cyclization was performed by dissolving the linear peptide in 10 mL of DCM/DMF 1:1 under continuous agitation. Then, DIPEA (2.4 equiv) was added and after 15 minutes HATU (1.2 equiv) and HOAt (1.2 equiv) were added, thus the resulting reaction mixture was stirred for 12 hours to afford the head-to-tail cyclized peptides (S1 Fig). At this point the side-chains protecting groups were removed using a 50% TFA solution in DCM (20 mL) for 2 h at rt. Then, the organic layer was evaporated by rotary evaporation under reduced pressure and the crude was recovered by precipitation in chilled diethyl ether, which was then removed to give a white powder. Crude peptides were finally purified by RP-HPLC using a semi-preparative C18-bonded silica column (Phenomenex, Jupiter 4μ Proteo 90Å, 1.0 x 25 cm) with a gradient of MeOH and water containing 0.1% TFA (from 0 to 90% over 40 min) at a flow rate of 5.0 mL/min. Products were obtained by lyophilization of the appropriate fractions after removal of the MeOH by rotary evaporation under reduced pressure. Analytical RP-HPLC indicated >95% purity and the correct molecular ions were confirmed by LC/ESI-MS.

### Serum stability

The stability of SRSRY and [SRSRY] was investigated in human serum. The peptides were incubated at 37°C in human serum at a concentration of 10^-2^mol/L. Aliquots (100 mL) of serum were removed at time points varying from 0, 1, 2, 4, 6, and 24 hours and acetonitrile (300 mL) was added to each aliquot before centrifuging (13000 rpm, 15 min). Aliquots (100 mL) of the supernatant were then analyzed by RP-HPLC after passing through Phenomenex Luna 100A C18 5μ, linear gradient from 0–90% acetonitrile in water over 25 minutes [41].

### Cell lines

Rat basophilic leukaemia RBL-2H3, and RBL-2H3/ETFR cells [25] were grown in DMEM-10% FBS. Human monocytic leukemia THP-1 cell line (purchased from the American Type Culture Collection), was cultured in RPMI 1640 medium, supplemented with 10% heat-inactivated fetal bovine serum (FBS), penicillin (100 μg/mL) and streptomycin (100 U/mL). Human umbilical vein endothelial cells (HUVECs), obtained from Lonza (C2519A, Lot # 0000115425), which provided a certificate of analysis for each cell lot, were grown in Eagle Basal Medium (EBM) supplemented with 4% FBS, 0.1% gentamicin, 1 μg/mL hydrocortisone, 10 μg/mL epidermal growth factor and 12 μg/mL bovine brain extract (Cambrex) [26]. All cells were maintained in an atmosphere of humidified air with 5% CO_2_ at 37°C.

### Differentiation and generation of GFP-transfected THP-1 cells

THP-1 differentiation into macrophages was induced using 160 nM phorbol-12-myristate acetate (PMA) purchased by Sigma Aldrich for 72 hours. To generate Green Fluorescent Protein (GFP)-tagged THP-1 cells, 2 x 10^7^ cells were re-suspended in 300 μL complete RPMI 1640 medium containing 20 mM Hepes and incubated with 5 μg pEGFP-N1 vector (Clontech) for 5 minutes on ice before being electroporated at 320 V, 1500 μF. Following electroporation, cells were kept on ice for 5 minutes then grown in 20 mL media for 24 hours. Then, G418-resistant cells expressing the highest levels of GFP were isolated and amplified.

### Preparation of human peripheral monocytes and macrophages

Blood was freshly drawn from healthy volunteers after informed written consent. Human peripheral blood mononuclear cells were harvested following centrifugation of whole blood on a density gradient solution composed of sodium metrizoate and dextran 500 (Lympholyte-poly Cell Separation Media, Cedarlane Laboratories) according to the manufacturer’s instructions. The plasma and mononuclear cells were individually collected. The pellet containing mononuclear cells was washed with PBS and transferred to tissue culture plates for 2 hours at 37°C with 5% CO_2_ to allow monocyte adherence. Plates were then gently washed to remove non-adherent cells, and monocytes were allowed to differentiate for 7 days in RPMI 1640 medium containing 10% autologous human serum.

### Ligand binding assay

RBL-2H3 cells, RBL-2H3/ETFR cells stable expressing FPR1, or THP-1 cells (1.5x10^6^cells/sample) were pre-incubated with diluents or the indicated unlabeled peptides for 60 minutes at 4°C (to avoid internalization), extensively rinsed with phosphate buffer saline (PBS), exposed to 10 nM N-formyl-Nle-Leu-Phe-Nle-Tyr-Lys-fluorescein (FITC-fMLF) purchased from Molecular Probes, diluted in PBS, for additional 60 minutes at 4°C and again rinsed with PBS. Quantification of cell-associated fluorescence was assessed by reading cells with a fluorescence plate reader Victor 3 (Perkin Elmer) using 485 nm excitation and 535 nm emission filters.

### Fluorescence microscopy

THP-1 cells (2x10^6^cells/sample) were incubated with the indicated unlabeled peptides for 30 minutes at 37°C and then exposed to 10 nM FITC-fMLF diluted in PBS for 30 minutes at 37°C as described [25]. To analyse cytoskeletal organization, PMA-differentiated THP-1 cells were fixed and permeabilized with 2.5% formaldehyde-0.1% Triton X-100 in PBS for 10 minutes at 4°C, washed in PBS and then incubated with 0.1 μg/mL rhodamine-conjugated phalloidin (Invitrogen) at 23°C for 45 minutes. In all cases, slides were mounted using 20% (w/v) mowiol, cells were visualized with an Axiovert 200M Inverted Fluorescent Microscope (Carl Zeiss) and images were taken with a videocamera.

### Cell migration assays

Migration of RBL-2H3/ETFR cells was analyzed using CIM-16-well plates and the xCELLigence RTCA technology. CIM plates are provided with interdigitated gold microelectrodes on bottom side of a filter membrane which is interposed between a lower and an upper compartment. The lower chamber was filled with 10 nM fMLF diluted in DMEM or growth medium with/without 10 nM [SRSRY]. Cells (3x10^4^ cells/well) were seeded on filters in serum-free medium. Microelectrodes detect impedance changes which are proportional to the number of migrating cells and are expressed as Cell Index. Migration was monitored in real-time for the indicated times. Each experiment was performed at least twice in quadruplicate. Migration of THP-1, PMA-stimulated THP-1 cells or primary monocytes was assessed in Boyden chambers (Neuroprobe) as described [15]. Cell suspension (1 x10^5^ viable cells per mL serum-free RPMI 1640 medium) was seeded in each upper chamber. Lower chambers were filled with RPMI 1640 medium containing 10 nM fMLF (Sigma-Aldrich), 10 nM SRSRY, as chemoattractants with/without [SRSRY] or [RSSYR], the last used as a scramble, control peptide. The two compartments were separated by an uncoated 5 μm pore size filter, in the case of THP-1 cells and primary monocytes, or by a collagen-coated (50 μg/mL for 2 hours at 37°C) 8 μm pore size polycarbonate filter (Neuroprobe) for PMA-stimulated THP-1 cells. Incubation, carried out at 37°C in humidified air with 5% CO_2_ was 90 minutes for THP-1 cells, and 3 hours for PMA-stimulated THP-1 cells. At the end of the assay, cells on the lower filter surface were fixed with ethanol, stained with haematoxylin and 10 random fields/filter were counted at 200x magnification.

### Cell proliferation

RBL-2H3/ETFR cell proliferation was assessed using E-16-well plates and the xCELLigence technology (Acea Bioscience, distributed by Roche Diagnostics) as described [12]. Briefly, cells (2x10^3^/well) were seeded in 16-well plates in growth medium and left to growth for 90 hours in the presence or the absence of 10 μM [SRSRY] or diluents. Microelectrodes placed on the bottom of plates, detect impedance changes which are proportional to the number of adherent cells and are expressed as Cell Index. The impedance value of each well was automatically monitored by the xCELLigence system and expressed as a Cell Index value. Growth medium with/ without [SRSRY] was replaced every 24 hours. The experiments were performed at least twice in quadruplicate.

### Dunn-chamber assay

PMA-stimulated THP-1 cells or primary macrophages were seeded on 20x20 mm coverslips for 24 hours. Before inverting the cover slip on top of a double concentric DUNN chamber, cells on the cover slip covering the outer chamber were carefully scraped away as previously described [25]. A gradient of a chemoattractant was created by placing serum-free medium in the inner chamber and 10 nM fMLF diluted in RPMI 1640, with/without peptides [SRSRY] or [RSSYR] in the outer chamber. The ring separating the inner and outer chambers permits slow diffusion between the chambers. For control experiments both wells were filled with serum-free medium. After 6 hours, the coverslip was removed from the chamber and the cytoskeleton was visualized by staining with rhodamine conjugated phalloidin. A total of 100 cells/sample that translocated to the area corresponding to the outer well were examined with a fluorescence-inverted microscope and images were taken with a videocamera.

### Monocyte diapedesis

Analysis of monocyte diapedesis was performed by seeding GFP-tagged THP-1 cells on an endothelial monolayer according to Ronald et al. [30]. To provide a substrate for monocyte migration, sterile round glass coverslips (12 mm in diameter) were coated with matrigel (Becton Dickinson) at a dilution of 1:8. The matrigel was air-dried at room temperature for 1 hour, followed by rehydration in EBM. HUVEC (5x10^4^ cells in 200 μL/well) were seeded onto matrigel and allowed to attach for 3 hours at 37°C, 5% CO_2_. Coverslips were then flooded with endothelial growth medium and incubated for at least 24 hours before the experiments Then, GFP-THP-1 cells (1x10^4^ cells/well) were exposed to diluents, or the indicated peptides in complete endothelial medium and added to endothelial monolayer at 37°C. After 30 minutes, slides were fixed, stained with rhodamine conjugated phalloidin and finally analyzed using an inverted fluorescence microscope (Axiovert 200,) or a confocal microscope (LSM510), both purchased by Carl Zeiss.

### Trans-endothelial migration assay

These assays were performed using E-16-well plates and the xCELLigence RTCA technology (Acea Bioscience) as described [12]. Microelectrodes placed on the bottom of plates, detect impedance changes which are proportional to the number of adherent cells. The impedance value of each well was automatically monitored by the xCELLigence system and expressed as a Cell Index value. HUVECs (1x10^4^ cells/well) suspended in growth medium, were seeded in E-16-well plates and allowed to grow for ~24 hours until they form a confluent monolayer, prior to seeding THP-1 cells (1x10^4^ cells/well) in growth medium plus/minus 10 nM [SRSRY]. When HUVECs are challenged with invading cells, there is a drop in electrical resistance within 2–10 hours which is monitored in real-time as the Cell Index changes due to invasion of the endothelial monolayer. The experiments were performed twice in quadruplicate.

### Statistical analysis

The results are expressed as the means ± SD of the number of the indicated determinations. Data were analyzed by one-way analysis of variance and post hoc Bonferonni’s modified t-test for multiple comparisons. p <0.01 was accepted as significant.

### Ethics statement

The research work with human blood samples has been approved by Institutional Ethical Committee of Istituto Nazionale Tumori “Fondazione G. Pascale”-IRCCS, Naples, Italy (protocol CEI 44/13).

## Supporting Information

S1 Fig Synthetic strategy. Cartoon showing the synthetic strategy employed to synthetize cyclic peptides. Details are included in the Materials and Methods.
(PDF)

S2 Fig Characterization of [SRSRY]: HPLC and Mass spectrum. SRSRY—Purity: 98.83%, t_R_: 7.603 (analytical HPLC, 0 to 90% MeOH in water (0.1%TFA) over 25 minutes, flow rate of 1.0 mL/min); molecular formula: C_27_H_46_N_12_O_8_, calculated mass: 666.26, API2000 ESIMS: *m*/*z* 667.36 [M + H]^+^; [SRSRY]—Purity: 95.41%, t_R_: 8.127 (analytical HPLC, 0 to 90% MeOH in water (0.1%TFA) over 25 minutes, flow rate of 1.0 mL/min); molecular formula: C_27_H_43_N_11_O_8_, calculated mass: 649.19, API2000 ESIMS: *m*/*z* 650.20 [M + H]^+^; [RSSYR]—Purity: 95.76%, t_R_: 8.512 (analytical HPLC, 0 to 90% MeOH in water (0.1%TFA) over 25 minutes, flow rate of 1.0 mL/min); molecular formula: C_27_H_43_N_11_O_8_, calculated mass: 649.19 API2000 ESIMS: *m*/*z* 650.20 [M + H]^+^.
(PDF)

S3 Fig Row data of Fig 2C. (PDF)

S4 Fig FBS-dependent RBL-2H3/ETFR cell proliferation in the presence of 10% FBS with or without 10 μM [SRSRY] Doubling times were calculated from the cell growth curves (time rangers: 5:12:47 ~ 24:13:25, 22:55:18 ~ 47:26:01 and 49:01:36 ~ 72:11:22). (PDF)

S5 Fig Row data of Fig 5D. (PDF)
