# Supplementary figures and images for: Cyclization of the Urokinase Receptor-Derived Ser-Arg-Ser-Arg-Tyr Peptide Generates a Potent Inhibitor of Trans-Endothelial Migration of Monocytes
Source: PLoS One. 2015 May 4;10(5):e0126172. doi: 10.1371/journal.pone.0126172 (PMC4418665; doi:10.1371/journal.pone.0126172)

**Figure S1**

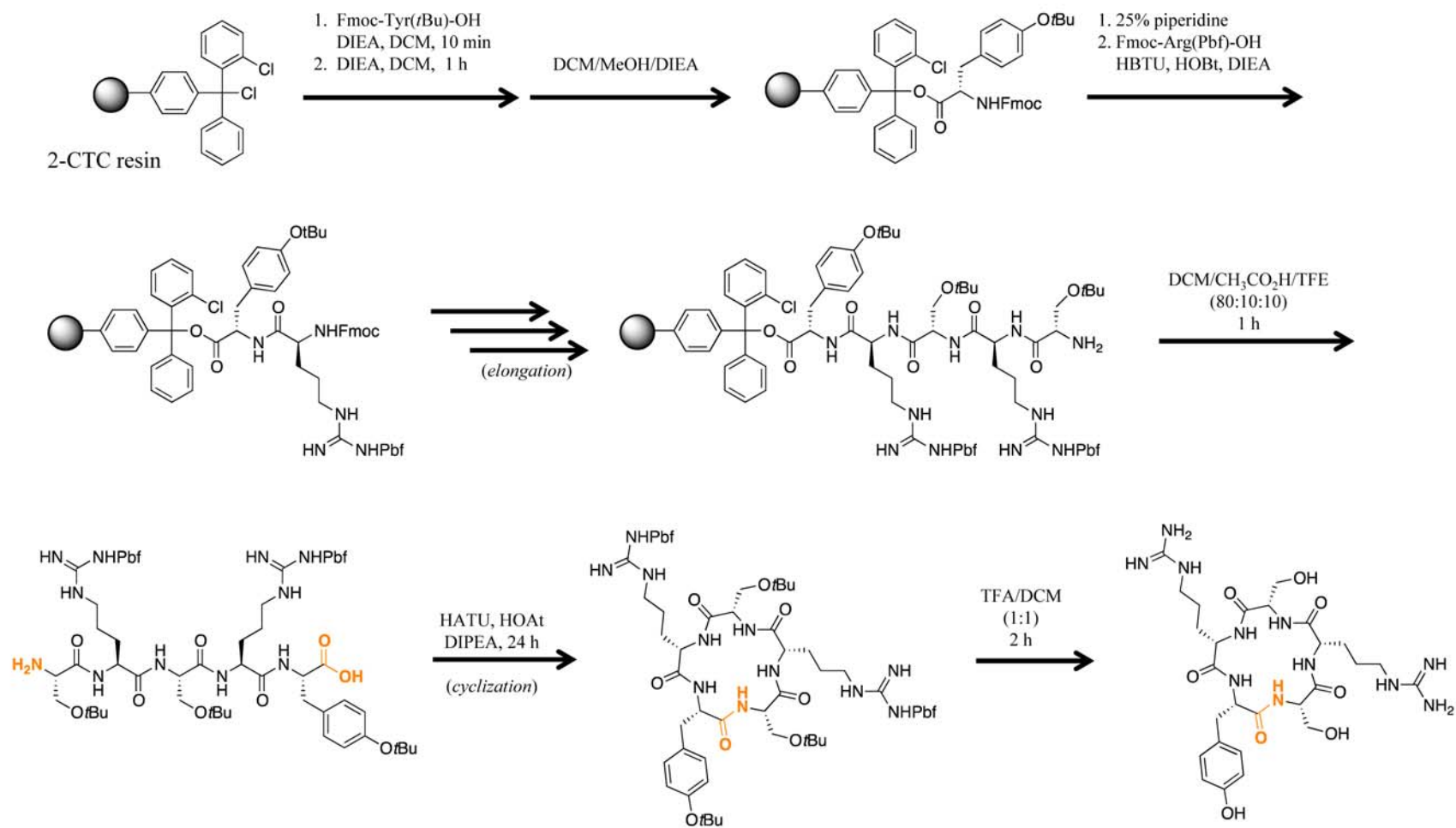

Supplement: S1 Fig — Cartoon showing the synthetic strategy employed to synthetize cyclic peptides. Details are included in the Materials and Methods. (PDF) [file pone.0126172.s001.pdf]

mAU

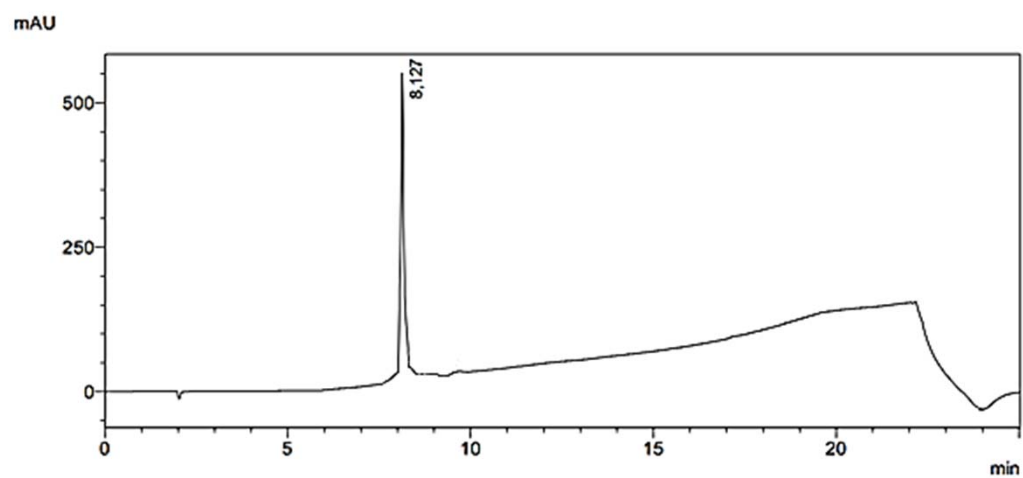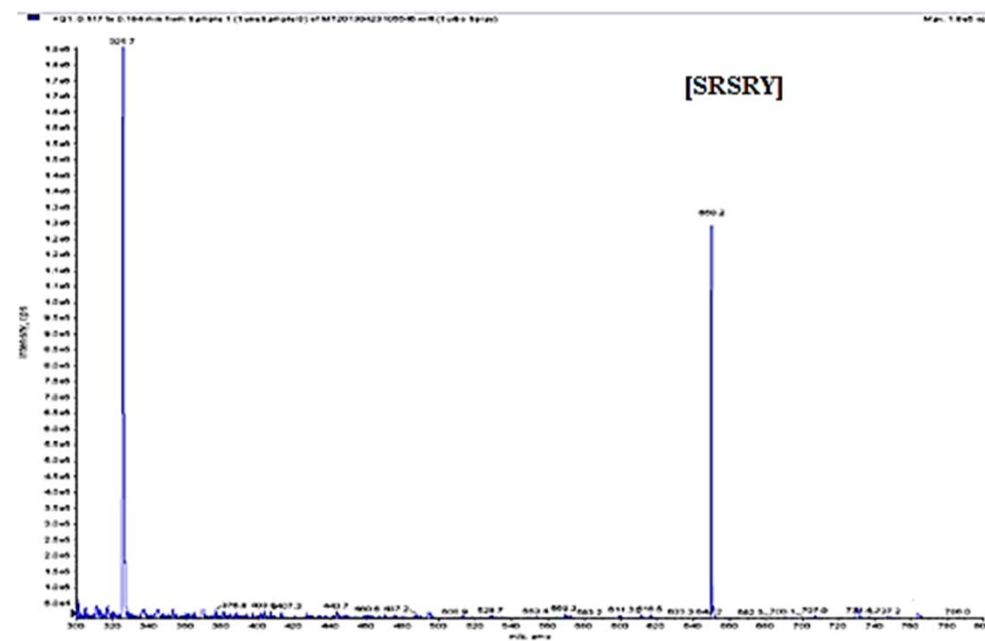

Supplement: S2 Fig — SRSRY—Purity: 98.83%, tR: 7.603 (analytical HPLC, 0 to 90% MeOH in water (0.1%TFA) over 25 minutes, flow rate of 1.0 mL/min); molecular formula: C27H46N12O8, calculated mass: 666.26, API2000 ESIMS: m/z 667.36 [M + H]+; [SRSRY]—Purity: 95.41%, tR: 8.127 (analytical HPLC, 0 to 90% MeOH in water (0.1%TFA) over 25 minutes, flow rate of 1.0 mL/min); molecular formula: C27H43N11O8, calculated mass: 649.19, API2000 ESIMS: m/z 650.20 [M + H]+; [RSSYR]—Purity: 95.76%, tR: 8.512 (analytical HPLC, 0 to 90% MeOH in water (0.1%TFA) over 25 minutes, flow rate of 1.0 mL/min); molecular formula: C27H43N11O8, calculated mass: 649.19 API2000 ESIMS: m/z 650.20 [M + H]+. (PDF) [file pone.0126172.s002.pdf]
