# Supplementary material for: Cyclization of the Urokinase Receptor-Derived Ser-Arg-Ser-Arg-Tyr Peptide Generates a Potent Inhibitor of Trans-Endothelial Migration of Monocytes
Source: PLoS One. 2015 May 4;10(5):e0126172. doi: 10.1371/journal.pone.0126172 (PMC4418665; doi:10.1371/journal.pone.0126172)
Supplement: S3 Fig — (PDF) [file pone.0126172.s003.pdf]

RBL-2H3/ETFR cell migration vs 10 nM [SRSRY] or
***vs* 10 nM fMLF +/-10 nM [SRSRY]**

	22/01/2014		12/02/2014		19/02/2014	
	CIM-Plate 1		CIM-Plate 2		CIM-Plate 3	
A	CTL	CTL	CTL	CTL	CTL	CTL
B	CTL	CTL	CTL	CTL	CTL	CTL
C	fMLF	fMLF	fMLF	fMLF	fMLF	fMLF
D	fMLF	fMLF	fMLF	fMLF	fMLF	fMLF
E	[SRSRY]	[SRSRY]	[SRSRY]	[SRSRY]	[SRSRY]	[SRSRY]
F	[SRSRY]	[SRSRY]	[SRSRY]	[SRSRY]	[SRSRY]	[SRSRY]
G	fMLF+ [SRSRY]	fMLF+ [SRSRY]	fMLF+ [SRSRY]	fMLF+ [SRSRY]	fMLF+ [SRSRY]	fMLF+ [SRSRY]
H	fMLF+ [SRSRY]	fMLF+ [SRSRY]	fMLF+ [SRSRY]	fMLF+ [SRSRY]	fMLF+ [SRSRY]	fMLF+ [SRSRY]

Cell Index at: 2:04:48 | Cell Index at: 2:04:48 | Cell Index at: 2:04:48

	1	2		1	2		1	2	*average*	*SD*
A	1,321	1,444	A	1,378	1,339	A	1,454	1,316	1,1912	0,227
B	0,833	1,065	B	0,858	1,16	B	0,895	1,231		
C	2,015	2,51	C	2,19	2,55	C	2,511	2,688	2,263	0,238
D	2,111	2,21	D	2,05	2,227	D	2,003	2,091		
E	1,266	1,438	E	1,353	1,532	E	1,433	1,583	1,3193	0,208
F	1,428	0,969	F	1,533	1,046	F	1,109	1,142		
G	1,45	1,236	G	1,531	1,61	G	1,582	1,585	1,3988	0,266
H	0,93	1,197	H	0,999	1,84	H	1,451	1,375		

P	
fMLF vs CTL	1,27145E-10
[SRSRY] vs CTL	6,49198E-10
fMLF+[SRSRY]v	0,423450564
fMLF+[SRSRY]v	2,6459E-08

Cell Index at: 2:54:46 | Cell Index at: 2:54:46 | Cell Index at: 2:54:46

	1	2		1	2		1	2				
A	1,676	1,377	A	1,737	1,442	A	1,602	1,503	1,4086	0,27	fMLF vs CTL	2,11819E-10
B	0,961	1,504	B	0,99	1,472	B	1,027	1,612				
C	2,494	2,31	C	2,49	2,68	C	2,511	2,488	2,5633	0,244	[SRSRY] vs CTL	4,19093E-12
D	2,777	3,1	D	2,498	2,637	D	2,6635	2,111				
E	1,588	1,505	E	1,561	1,566	E	1,537	1,698	1,4965	0,126	fMLF+[SRSRY]v	0,298456609
F	1,517	1,241	F	1,587	1,414	F	1,339	1,405				
G	1,649	1,614	G	1,618	1,676	G	1,673	1,655	1,5502	0,121	fMLF+[SRSRY]v	1,0016E-11
H	1,347	1,503	H	1,339	1,578	H	1,499	1,451				

Cell Index at: 4:04:46			Cell Index at: 4:04:46			Cell Index at: 4:04:46						
	1	2		1	2		1	2				
A	1,507	1,774	A	1,559	1,806	A	1,772	1,643	1,5151	0,263	fMLF vs CTL	7,16219E-12
B	1,213	1,685	B	1,213	1,327	B	1,012	1,67				
C	2,594	2,944	C	2,994	3,314	C	3,599	2,944	3,1102	0,33	[SRSRY] vs CTL	1,29099E-12
D	2,798	3,157	D	2,898	3,157	D	3,766	3,157				
E	1,604	1,593	E	1,639	1,65	E	1,178	1,711	1,5718	0,174	fMLF+[SRSRY]v	0,40198669
F	1,671	1,378	F	1,614	1,51	F	1,457	1,857				
G	1,721	1,473	G	1,666	2,099	G	1,789	1,761	1,637	0,199	fMLF+[SRSRY]v	5,67614E-12
H	1,464	1,336	H	1,522	1,681	H	1,588	1,544				
