# Supplementary material for: Cyclization of the Urokinase Receptor-Derived Ser-Arg-Ser-Arg-Tyr Peptide Generates a Potent Inhibitor of Trans-Endothelial Migration of Monocytes
Source: PLoS One. 2015 May 4;10(5):e0126172. doi: 10.1371/journal.pone.0126172 (PMC4418665; doi:10.1371/journal.pone.0126172)

FBS-dependent RBL-2H3/ETFR cell proliferation in the presence of 10%FBS with or without 10 µM [SRSRY]

	**10/11/2014**		**24/11/2014**	
	**E-Plate 1**		**E-Plate 2**	
A	**FBS**	**FBS**	**FBS**	**FBS**
B	**FBS**	**FBS**	**FBS**	**FBS**
C	**FBS + 10 µM [SRSRY]**	**FBS + 10 µM [SRSRY]**	**FBS + 10 µM [SRSRY]**	**FBS + 10 µM [SRSRY]**
D	**FBS + 10 µM [SRSRY]**	**FBS + 10 µM [SRSRY]**	**FBS + 10 µM [SRSRY]**	**FBS + 10 µM [SRSRY]**

Doubling time: 5:12:47 ~ 24:13:25

Doubling time: 22:55:18 ~ 47:26:01

Doubling time: 49:01:36 ~ 72:11:22

		Time			SD		
		5:12:47 ~ 24:13:25	22:55:18 ~ 47:26:01	49:01:36 ~ 72:11:22	5:12:47 ~ 24:13:25	5:18 ~ 47:2	1:36 ~ 72:11:22
E-Plate 1	FBS	41,1233	21,692	72,1523	1,0184	0,1238	1,5078
E-Plate 2	FBS	41,1851	21,4601	76,4721	0,9328	0,213	1,5558
E-Plate 1	FBS + 10µM [SRSRY]	39,06568	22,9396	65,9629	1,1465	0,1257	2,5048
E-Plate 2	FBS + 10µM [SRSRY]	38,5601	23,0289	64,2637	1,1284	0,1809	1,3611

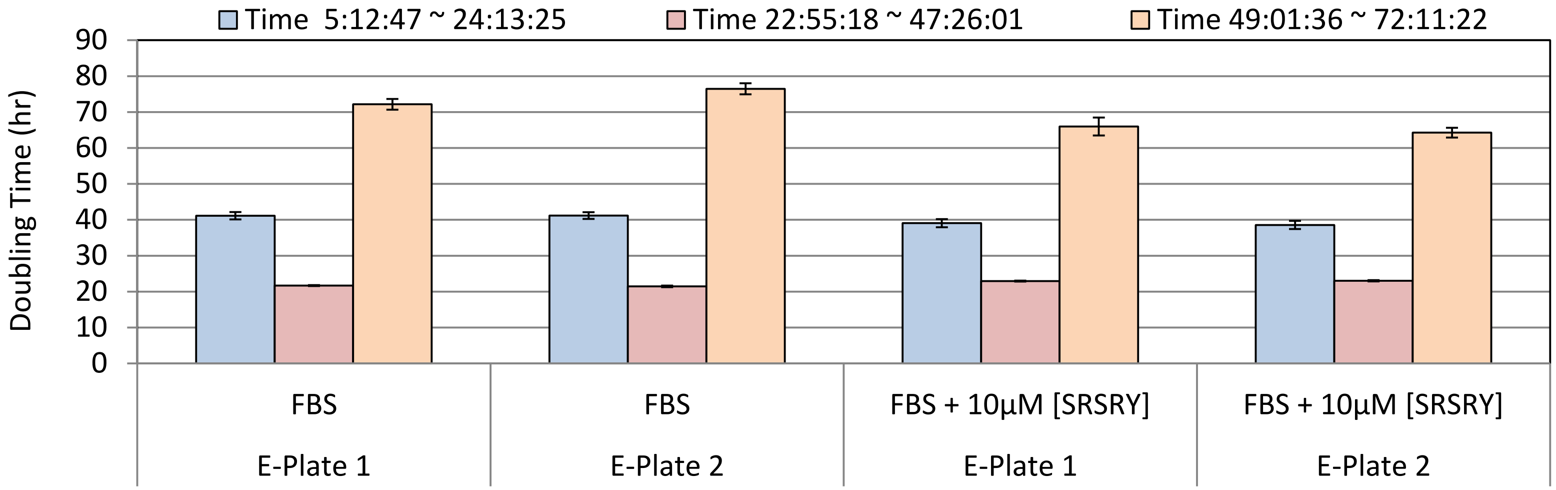

Supplement: S4 Fig — (PDF) [file pone.0126172.s004.pdf]
