# Supplementary material for: Cyclization of the Urokinase Receptor-Derived Ser-Arg-Ser-Arg-Tyr Peptide Generates a Potent Inhibitor of Trans-Endothelial Migration of Monocytes
Source: PLoS One. 2015 May 4;10(5):e0126172. doi: 10.1371/journal.pone.0126172 (PMC4418665; doi:10.1371/journal.pone.0126172)
Supplement: S5 Fig — (PDF) [file pone.0126172.s005.pdf]

Endothelial invasion by THP-1 cells

	25/06/2014 E-Plate 1			02/07/2014 E-Plate 2	
A	[SRSRY]	[SRSRY]		[SRSRY]	[SRSRY]
B	[SRSRY]	[SRSRY]		[SRSRY]	[SRSRY]
C	None	None		None	None
D	None	None		None	None
G	HUVEC	HUVEC		HUVEC	HUVEC
H	HUVEC	HUVEC		HUVEC	HUVEC

Cell Index at: 25:44:44

	1	2
A	1,51744737	1,493789
B	1,49439474	1,376816
C	1,01599624	0,978853
D	1,04996241	1,092669
G	1,21160526	1,353868
H	1,39594737	1,2955

Cell Index at: 25:44:44

	1	2
A	1,57613158	1,581132
B	1,60073684	1,471158
C	1,0831391	1,055602
D	1,12733083	1,177575
G	1,34342105	1,524816
H	1,52581579	1,407421

Cell Index at: 26:04:34

	1	2
A	1,64713158	1,655974
B	1,71502632	1,584368
C	1,1412406	1,150733
D	1,22545113	1,276767
G	1,53547368	1,751079
H	1,66355263	1,537105

Cell Index at: 26:04:34

	1	2
A	1,64713158	1,655974
B	1,71502632	1,584368
C	1,1412406	1,150733
D	1,22545113	1,276767
G	1,53547368	1,751079
H	1,66355263	1,537105

Cell Index at: 26:24:24

	1	2
A	1,53497368	1,516737
B	1,54110526	1,457947
C	1,07007519	1,073759
D	1,12204887	1,186748
G	1,49286842	1,675711
H	1,56697368	1,446132

Cell Index at: 26:24:24

	1	2
A	1,49323684	1,469658
B	1,48802632	1,418105
C	1,04593985	1,039492
D	1,08847744	1,148778
G	1,46626316	1,648868
H	1,5335	1,420711

Cell Index at: 26:43:46

	1	2
A	1,40460526	1,388342
B	1,37647368	1,340079
C	0,98968045	0,98391

Cell Index at: 26:43:46

	1	2
A	1,37786842	1,363789
B	1,35657895	1,323211
C	0,98110902	0,970451

D	1,0137406	1,063966
G	1,42342105	1,584237
H	1,47297368	1,372368

Cell Index at: 27:03:30

	1	2
A	1,33636842	1,329921
B	1,32902632	1,295789
C	0,96488722	0,951635
D	0,98332707	1,012707
G	1,27681818	1,436675
H	1,32801435	1,24067

Cell Index at: 27:23:30

	1	2
A	1,31828947	1,310263
B	1,31007895	1,287237
C	0,93725564	0,947632
D	0,98419173	0,998402
G	1,30093301	1,463469
H	1,33837321	1,253708

Cell Index at: 27:43:30

	1	2
A	1,28381579	1,268316
B	1,27942105	1,239605
C	0,90503759	0,92532
D	0,96821429	0,977876
G	1,31255981	1,471699
H	1,34019139	1,258254

Cell Index at: 28:03:30

	1	2
A	1,24007895	1,219816
B	1,23544737	1,183211
C	0,86477444	0,892143
D	0,93892857	0,950357
G	1,31244019	1,463636
H	1,32739234	1,248517

Cell Index at: 28:23:26

	1	2
A	1,18173684	1,157526
B	1,17223684	1,126368
C	0,83642857	0,859812
D	0,9049812	0,906466

D	0,99870301	1,04094
G	1,41736842	1,575553
H	1,45921053	1,371079

Cell Index at: 27:03:30

	1	2
A	1,33531579	1,323395
B	1,32552632	1,286421
C	0,96129699	0,952688
D	0,98058271	1,011786
G	1,28248804	1,441555
H	1,33526316	1,241388

Cell Index at: 27:23:30

	1	2
A	1,31260526	1,305526
B	1,30297368	1,276421
C	0,92796992	0,942914
D	0,98011278	0,992951
G	1,30248804	1,470048
H	1,33854067	1,255359

Cell Index at: 27:43:30

	1	2
A	1,27092105	1,260079
B	1,26673684	1,226921
C	0,89928571	0,916598
D	0,9606203	0,972782
G	1,30942584	1,473517
H	1,34066986	1,255789

Cell Index at: 28:03:30

	1	2
A	1,22760526	1,201263
B	1,21552632	1,165184
C	0,85646617	0,883722
D	0,93242481	0,939699
G	1,30842105	1,461388
H	1,32607656	1,24866

Cell Index at: 28:23:26

	1	2
A	1,17007895	1,142158
B	1,16326316	1,114263
C	0,83065789	0,846353
D	0,89821429	0,897782

G	1,28366029	1,452703		G	1,27964115	1,44689
H	1,31528708	1,228014		H	1,3107177	1,226172

Cell Index at: 28:43:26 | Cell Index at: 28:43:26

	1	2			1	2
A	1,13528947	1,098316		A	1,12407895	1,085789
B	1,11392105	1,070789		B	1,10076316	1,056632
C	0,80577068	0,820338		C	0,79917293	0,809361
D	0,86471805	0,862462		D	0,85287594	0,852312
G	1,26399522	1,420287		G	1,2619378	1,413589
H	1,29258373	1,202201		H	1,28818182	1,191053

Cell Index at: 29:03:26 | Cell Index at: 29:03:26

	1	2			1	2
A	1,00196172	0,963421		G	0,99322967	0,954737
B	0,9722488	0,934211		H	0,96110048	0,925574
C	0,77902256	0,783195		C	0,77150376	0,778571
D	0,82924812	0,830996		D	0,82477444	0,823064
G	1,37928947	1,531421		G	1,37565789	1,530132
H	1,41415789	1,294105		H	1,41128947	1,291842

Cell Index at: 29:23:26 | Cell Index at: 29:23:26

	1	2			1	2
A	0,96904306	0,93677		G	0,96208134	0,931555
B	0,93566986	0,906196		H	0,93205742	0,899163
C	0,75578947	0,765376		C	0,75005639	0,763214
D	0,81524436	0,811071		D	0,81112782	0,79953
G	1,37236842	1,521553		G	1,36760526	1,519316
H	1,40023684	1,282711		H	1,39934211	1,279553

Cell Index at: 29:43:26 | Cell Index at: 29:43:26

	1	2			1	2
A	0,94964115	0,90811		G	0,94964115	0,90811
B	0,91583732	0,876603		H	0,91583732	0,876603
C	0,74022556	0,750244		C	0,74022556	0,750244
D	0,79853383	0,779774		D	0,79853383	0,779774
G	1,36842105	1,519026		G	1,36842105	1,519026
H	1,38778947	1,266816		H	1,38778947	1,266816

Cell Index at: 30:03:26 | Cell Index at: 30:03:26

	1	2			1	2
A	0,92732057	0,884474		G	0,92485646	0,880096
B	0,9073445	0,870263		H	0,90703349	0,867943
C	0,72590226	0,733778		C	0,72071429	0,729211
D	0,78137218	0,771617		D	0,77823308	0,769586
G	1,37107895	1,524947		G	1,37342105	1,520395

H	1,39292105	1,268711

H	1,39881579	1,273237

Cell Index at: 30:23:26

	1	2
A	0,91136364	0,865694
B	0,8961244	0,850335
C	0,71716165	0,719812
D	0,7675188	0,76844
G	1,37031579	1,5195
H	1,39694737	1,272158

Cell Index at: 30:23:26

	1	2
G	0,90811005	0,862488
H	0,88913876	0,844761
C	0,71518797	0,717932
D	0,76129699	0,763985
G	1,36471053	1,518368
H	1,39713158	1,276289

Cell Index at: 30:43:26

	1	2
A	0,90794258	0,850167
B	0,88406699	0,827464
C	0,70703008	0,709586
D	0,7562406	0,75438
G	1,24205742	1,373301
H	1,26514354	1,158158

Cell Index at: 30:43:26

	1	2
G	0,90052632	0,844258
H	0,88291866	0,826746
C	0,70383459	0,703665
D	0,74879699	0,748064
G	1,24021531	1,373325
H	1,2676555	1,155526

Cell Index at: 31:03:26

	1	2
A	0,89172249	0,828708
B	0,8776555	0,82134
C	0,69667293	0,694643
D	0,7425	0,739944
G	1,36771053	1,508342
H	1,38902632	1,265947

Cell Index at: 31:03:26

	1	2
G	0,88641148	0,829163
H	0,87839713	0,815215
C	0,69631579	0,690996
D	0,73757519	0,736034
G	1,36965789	1,503974
H	1,38631579	1,265868

Cell Index at: 31:23:26

	1	2
A	0,874689	0,821555
B	0,86954545	0,811722
C	0,69161654	0,675883
D	0,72721805	0,724248
G	1,24095694	1,366459
H	1,26045455	1,150813

Cell Index at: 31:23:26

	1	2
G	0,87492823	0,820742
H	0,8673445	0,810215
C	0,68968045	0,670959
D	0,71996241	0,717293
G	1,23988038	1,366459
H	1,25488038	1,150909

Cell Index at: 31:43:26

	1	2
A	0,87126794	0,807536
B	0,85980861	0,807823
C	0,68406015	0,662556
D	0,71406015	0,714079
G	1,24244019	1,367344
H	1,2672488	1,151914

Cell Index at: 31:43:26

	1	2
G	0,8677512	0,800455
H	0,85284689	0,804952
C	0,68144737	0,660789
D	0,70941729	0,713365
G	1,23875598	1,363062
H	1,26722488	1,157081

Cell Index at: 32:03:26

	1	2
A	0,86270335	0,788923
B	0,83995215	0,792871
C	0,67441729	0,652256
D	0,70456767	0,705226
G	1,23227273	1,365383
H	1,27095694	1,152823

Cell Index at: 32:03:26

	1	2
G	0,85028708	0,787799
H	0,83961722	0,786962
C	0,67214286	0,649154
D	0,70402256	0,702293
G	1,22923445	1,368182
H	1,27105263	1,151507

Cell Index at: 32:23:26

	1	2
A	0,8358134	0,776411
B	0,83014354	0,777727
C	0,66652256	0,641654
D	0,69556391	0,69282
G	1,23382775	1,372703
H	1,26880383	1,151938

Cell Index at: 32:23:26

	1	2
G	0,82937799	0,771842
H	0,83050239	0,773254
C	0,66276316	0,636241
D	0,69610902	0,688008
G	1,22980861	1,362177
H	1,26858852	1,154402

Cell Index at: 32:43:26

	1	2
A	0,78173913	0,722609
B	0,78526316	0,733547
C	0,65802632	0,627387
D	0,68460526	0,68344
G	1,23397129	1,346268
H	1,25952153	1,147799

Cell Index at: 32:43:26

	1	2
G	0,77544622	0,717757
H	0,77926773	0,724508
C	0,65428571	0,627425
D	0,68556391	0,678609
G	1,23401914	1,348469
H	1,25808612	1,150431

Cell Index at: 33:03:26

	1	2
A	0,76798627	0,704233
B	0,76704805	0,714279
C	0,64830827	0,619549
D	0,67966165	0,669906
G	1,24272727	1,339809
H	1,25382775	1,148804

Cell Index at: 33:03:26

	1	2
G	0,76464531	0,696293
H	0,76640732	0,712975
C	0,6456391	0,61515
D	0,67601504	0,668365
G	1,24253589	1,339402
H	1,25327751	1,149665

Cell Index at: 33:23:26

	1	2
A	0,76475973	0,685744
B	0,75750572	0,705011
C	0,63731203	0,607068
D	0,66479323	0,667895
G	1,23947368	1,334856
H	1,2562201	1,14488

Cell Index at: 33:23:26

	1	2
G	0,76118993	0,680366
H	0,75649886	0,705675
C	0,63434211	0,60594
D	0,66490602	0,664023
G	1,23746411	1,33311
H	1,25636364	1,141699

Cell Index at: 33:43:26

	1	2
A	0,75343249	0,663867
B	0,74114416	0,702105
C	0,63073308	0,598872
D	0,65774436	0,647989
G	1,23641148	1,335646
H	1,2480622	1,135311

Cell Index at: 33:43:26

	1	2
G	0,75290618	0,663364
H	0,73750572	0,699931
C	0,63018797	0,598083
D	0,65612782	0,644286
G	1,23894737	1,331699
H	1,24660287	1,133206

Cell Index at: 34:03:26

	1	2
A	0,73853547	0,650114
B	0,72665904	0,691533
C	0,61798872	0,592462
D	0,64650376	0,623496
G	1,23837321	1,329234
H	1,24342105	1,125646

Cell Index at: 34:03:26

	1	2
G	0,73736842	0,647483
H	0,72629291	0,687368
C	0,61208647	0,590677
D	0,64492481	0,617857
G	1,24062201	1,325024
H	1,24004785	1,122392

Cell Index at: 34:23:26

	1	2
A	0,72954233	0,63897
B	0,71654462	0,676888
C	0,60464286	0,585489
D	0,64462406	0,60938
G	1,22825359	1,321459
H	1,23944976	1,118923

Cell Index at: 34:23:26

	1	2
G	0,72540046	0,635606
H	0,71574371	0,673776
C	0,6037218	0,585207
D	0,64236842	0,607274
G	1,22528708	1,322847
H	1,23789474	1,114545

Cell Index at: 34:43:26

	1	2
A	0,67949561	0,604693
B	0,67203947	0,638596
C	0,6012782	0,574342
D	0,63118421	0,600094
G	1,21956938	1,319665
H	1,23220096	1,115957

Cell Index at: 34:43:26

	1	2
G	0,67497807	0,603465
H	0,66848684	0,633991
C	0,59740602	0,571259
D	0,62834586	0,601203
G	1,21827751	1,318373
H	1,23327751	1,114019

Cell Index at: 35:03:26

	1	2
A	0,66085526	0,597325
B	0,65754386	0,631294
C	0,58308271	0,567538
D	0,62009398	0,593289
G	1,21334928	1,319211
H	1,22770335	1,117488

Cell Index at: 35:03:26

	1	2
A	0,79055263	0,714658
B	0,78226316	0,753605
C	0,58394737	0,566203
D	0,61592105	0,589192
G	1,21409091	1,315239
H	1,2280622	1,117488

Cell Index at: 35:23:26

	1	2
A	0,65589912	0,589276
B	0,64366228	0,622281
C	0,58351504	0,562594
D	0,61011278	0,579154
G	1,21043062	1,30811
H	1,2138756	1,116005

Cell Index at: 35:23:26

	1	2
A	0,78602632	0,704026
B	0,77326316	0,742632
C	0,58067669	0,561071
D	0,50539474	0,575282
G	1,20868421	1,306124
H	1,21186603	1,113708

Cell Index at: 35:43:26

	1	2
A	0,64739035	0,578596
B	0,64089912	0,620702
C	0,69960526	0,780553
D	0,5968797	0,567519
G	1,21076555	1,290455
H	1,21062201	1,108995

Cell Index at: 35:43:26

	1	2
A	0,76989474	0,693632
B	0,76865789	0,740211
C	0,59628947	0,579421
D	0,59285714	0,566015
G	1,20925837	1,2889
H	1,21547847	1,103732
